# pH-responsive hierarchical H_2_S-releasing nano-disinfectant with deep-penetrating and anti-inflammatory properties for synergistically enhanced eradication of bacterial biofilms and wound infection

**DOI:** 10.1186/s12951-022-01262-7

**Published:** 2022-01-29

**Authors:** Yue Zhang, Tianxiang Yue, Wenting Gu, Aidi Liu, Mengying Cheng, Hongyue Zheng, Dandan Bao, Fanzhu Li, Ji-Gang Piao

**Affiliations:** 1 School of Pharmaceutical Sciences, Zhejiang Chinese Medical University, Hangzhou, 310053 People’s Republic of China; 2 Key Laboratory of Neuropharmacology and Translational Medicine of Zhejiang Province, School of Pharmaceutical Sciences, Zhejiang Chinese Medical University, Hangzhou, 310053 People’s Republic of China; 3 Libraries of Zhejiang Chinese Medical University, Zhejiang Chinese Medical University, Hangzhou, 310053 People’s Republic of China; 4 Department of Dermatology & Cosmetology, The First Affiliated Hospital of Zhejiang Chinese Medical University (Zhejiang Provincial Hospital of Traditional Chinese Medicine), Hangzhou, 310053 People’s Republic of China; 5 Academy of Chinese Medical Science, Zhejiang Chinese Medical University, Hangzhou, 310053 People’s Republic of China

**Keywords:** Anti-biofilm, Zinc sulfide, Hydrogen sulfide, Gas therapy, Hyperthermia therapy, MRSA, Penetration

## Abstract

**Background:**

Methicillin-resistant *Staphylococcus aureus* (MRSA) biofilm-associated bacterial infection is the primary cause of nosocomial infection and has long been an ongoing threat to public health. MRSA biofilms are often resistant to multiple antimicrobial strategies, mainly due to the existence of a compact protective barrier; thus, protecting themselves from the innate immune system and antibiotic treatment via limited drug penetration.

**Results:**

A hierarchically structured hydrogen sulfide (H_2_S)-releasing nano-disinfectant was presented, which was composed of a zinc sulfide (ZnS) core as a H_2_S generator and indocyanine green (ICG) as a photosensitizer. This nano-disinfectant (ICG-ZnS NPs) sensitively responded to the biofilm microenvironment and demonstrated efficient eradication of MRSA biofilms via a synergistic effect of Zn^2+^, gas molecule-mediated therapy, and hyperthermia. Physically boosted by released H_2_S and a near-infrared spectroscopy-induced hyperthermia effect, ICG-ZnS NPs destroyed the compactness of MRSA biofilms showing remarkable deep-penetration capability. Moreover, on-site generation of H_2_S gas adequately ameliorated excessive inflammation, suppressed secretion of inflammatory cytokines, and expedited angiogenesis, therefore markedly accelerating the in vivo healing process of cutaneous wounds infected with MRSA biofilms.

**Conclusion:**

ICG-ZnS NPs combined with NIR laser irradiation exhibited significant anti-biofilm activity in MRSA biofilms, can accelerate the healing process through deep-penetration and anti-inflammatory effectuation. The proposed strategy has great potential as an alternative to antibiotic treatment when combating multidrug-resistant bacterial biofilms.

**Graphical Abstract:**

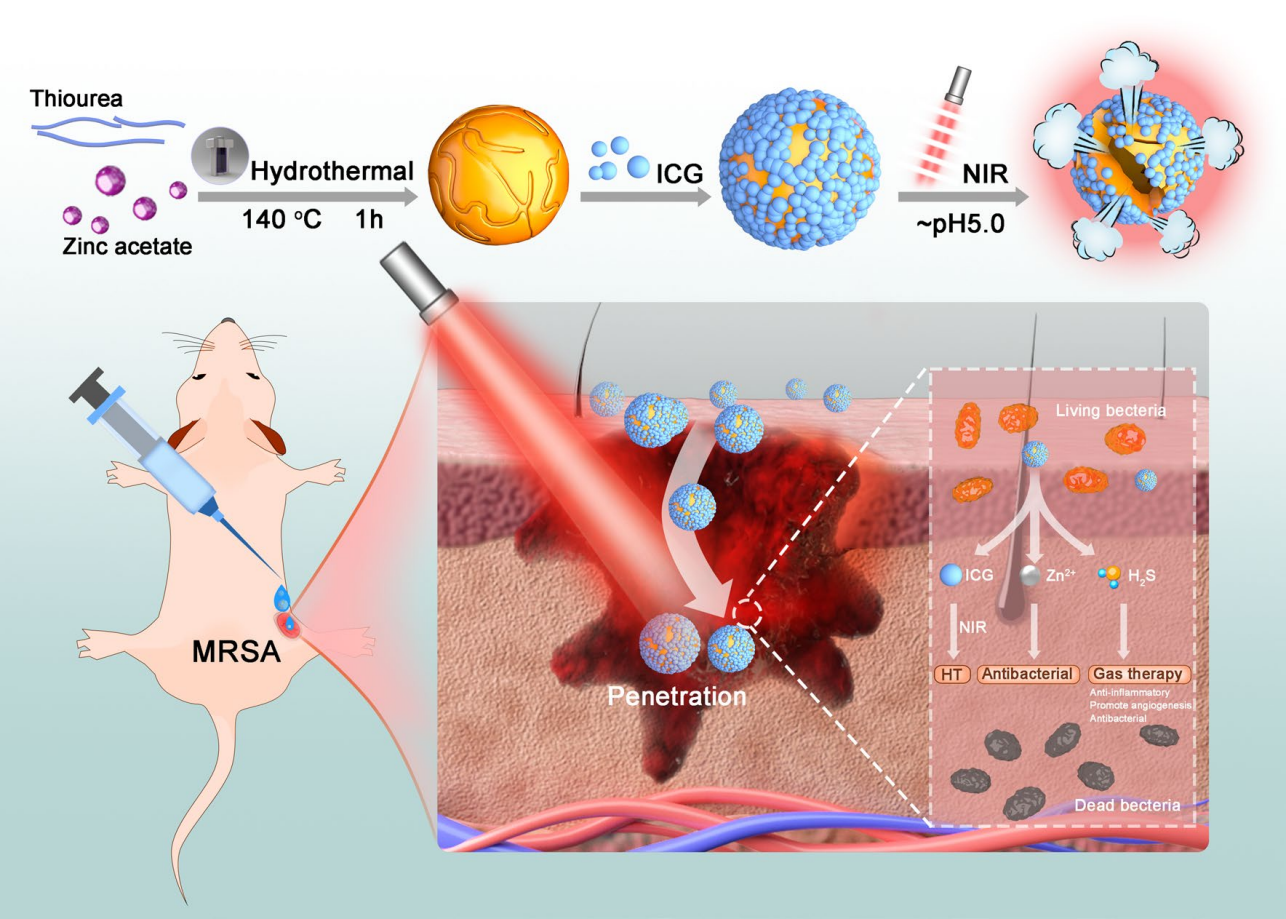

**Supplementary Information:**

The online version contains supplementary material available at 10.1186/s12951-022-01262-7.

## Introduction

The incremental emergence of antibiotic resistance remains a global health issue [1, 2], estimated to cause the loss of 10 million lives by the year 2050 [3]. In particular, methicillin-resistant *Staphylococcus aureus* (MRSA) biofilm-associated nosocomial infections have become the most critical threat to public health [4–6]. The latest data regarding MRSA incidence acquired from over 80 countries shows that the mortality rates in the past years were still high with values exceeding 20% [7]. MRSA biofilms are protective communities that are enclosed in self-generated extracellular polymeric substances [8, 9], that functionalizes as a defensive barrier by limiting the inward penetration of antimicrobials [10], inducing localized inflammation [11], and obstructing angiogenesis [12]. Currently, there is still lacking effective therapeutic modalities to counteract the as-formed MRSA biofilms while ameliorating the associated inflammatory responses, demonstrating an urgent requirement for novel approaches for effective treatments in anti-biofilm applications [13–15].

Gas molecule-mediated therapy (GT) features high diffusibility and penetrability and has shown broad prospects for biomedical applications [16–19]. Hydrogen sulfide (H_2_S), as the third biological gasotransmitter following carbon monoxide and nitric oxide, plays various physiological roles, including regulating ion channels, modulating inflammatory responses [20, 21], and promoting angiogenesis [22]. Previous studies have demonstrated that H_2_S is a biofilm disruptor and is considered as a latent way to permeate MRSA biofilms [23]. It can also increase blood perfusion to the wound, which may contribute to bacterial infection clearance and the restorative process acceleration by activating ATP-sensitive potassium channels to increase neutrophil migration [24]. Nevertheless, due to the Janus-faced pharmacological character of H_2_S, lack of controllability and targeting specificity remains a challenge for biomedical application of H_2_S.

Hyperthermia therapy (HT) has been proven an efficient and skin-safe therapeutic modality to combat biofilms, with therapeutic effects in the temperature range of 40–45 °C [25–27]. Besides the intrinsic damage to bacteria caused by the localized mild heat, the hyperthermia effect also increases blood flow and induces vascular endothelial cell proliferation via mimicking the “hot spring effect”, thus accelerating wound healing [28]. Therefore, an appealing strategy is to integrate HT with GT for dealing with the issue of MRSA biofilms. Thanks to the enormous advance in nanotechnology during the past decades, which offers more options for on-demand delivery of therapeutic agents to pathological sites with enhanced targeting ability [5, 29]. With advantages of controlled release, high efficiency, and low toxicity, pH-triggered H_2_S-generating nanomaterials targeting biofilm microenvironment (BME) have attracted widespread interest [30–32]. Zinc sulfide nanoparticles (ZnS NPs), a novel metallic chalcogenides nanomaterial of class II-IV semiconductors with promising antibacterial and re-epithelization-enhancing activities, can be degraded in acidic microenvironments and generate H_2_S gas [33, 34]. The released Zn^2+^ can restrain enzymatic activity and prevent cell metabolism above the ion concentration threshold [26]. Moreover, Zn^2+^ synergizes with H_2_S to depolarize the bacterial cell membrane [35], making ZnS NPs an ideal candidate as nano-disinfectant and integration nanoplatform for eliminating MRSA biofilms.

**Fig. 1:**
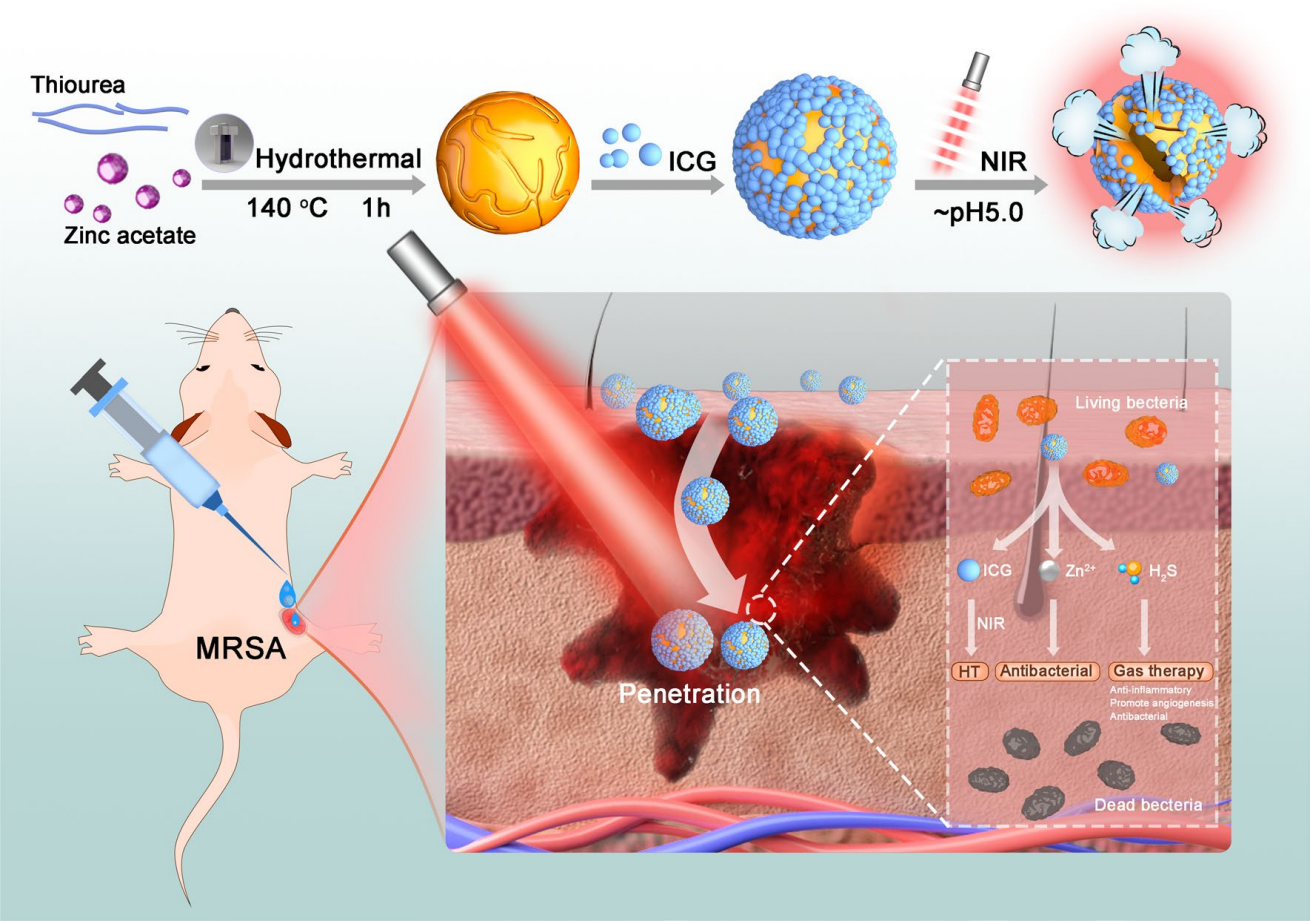
Schematic illustration of the composition and the antibacterial mechanism of biofilm-responsive hierarchical H_2_S-releasing nano-disinfectant for synergistically enhanced eradication of MRSA biofilms and wound infection

Inspired by this, we hypothesized that combining ZnS NPs with the photosensitizer Indocyanine green (ICG, an FDA-approved near-infrared (NIR) organic dye) (ICG-ZnS NPs) would be an effective therapeutic strategy concomitantly for NIR-mediated HT and GT to rapidly realize the healing of MRSA-biofilm-infected wounds [36–38]. Upon entering the acidic BME, ICG-ZnS NPs released Zn^2+^ and H_2_S, specifically destroying compactness of MRSA biofilms (Fig. 1). Furthermore, physically boosted by released H_2_S and the NIR-induced hyperthermia effect, ICG-ZnS NPs destroyed the compactness of the MRSA biofilms and depolarized the bacterial cell membrane, showing remarkable deep-penetration capability. On-site H_2_S gas generation adequately ameliorated excessive inflammation, suppressed inflammatory cytokine secretion, and expedited angiogenesis, therefore markedly accelerating the healing process of cutaneous wounds infected with MRSA biofilms, in a relatively bio-safe way by combining HT with H_2_S and Zn^2+^. This attempt to utilize a GT-HT combination on bacterial infections shows great promise for further applications.

## Results and discussion

### Preparation and characterization of ICG-ZnS NPs

**Fig. 2:**
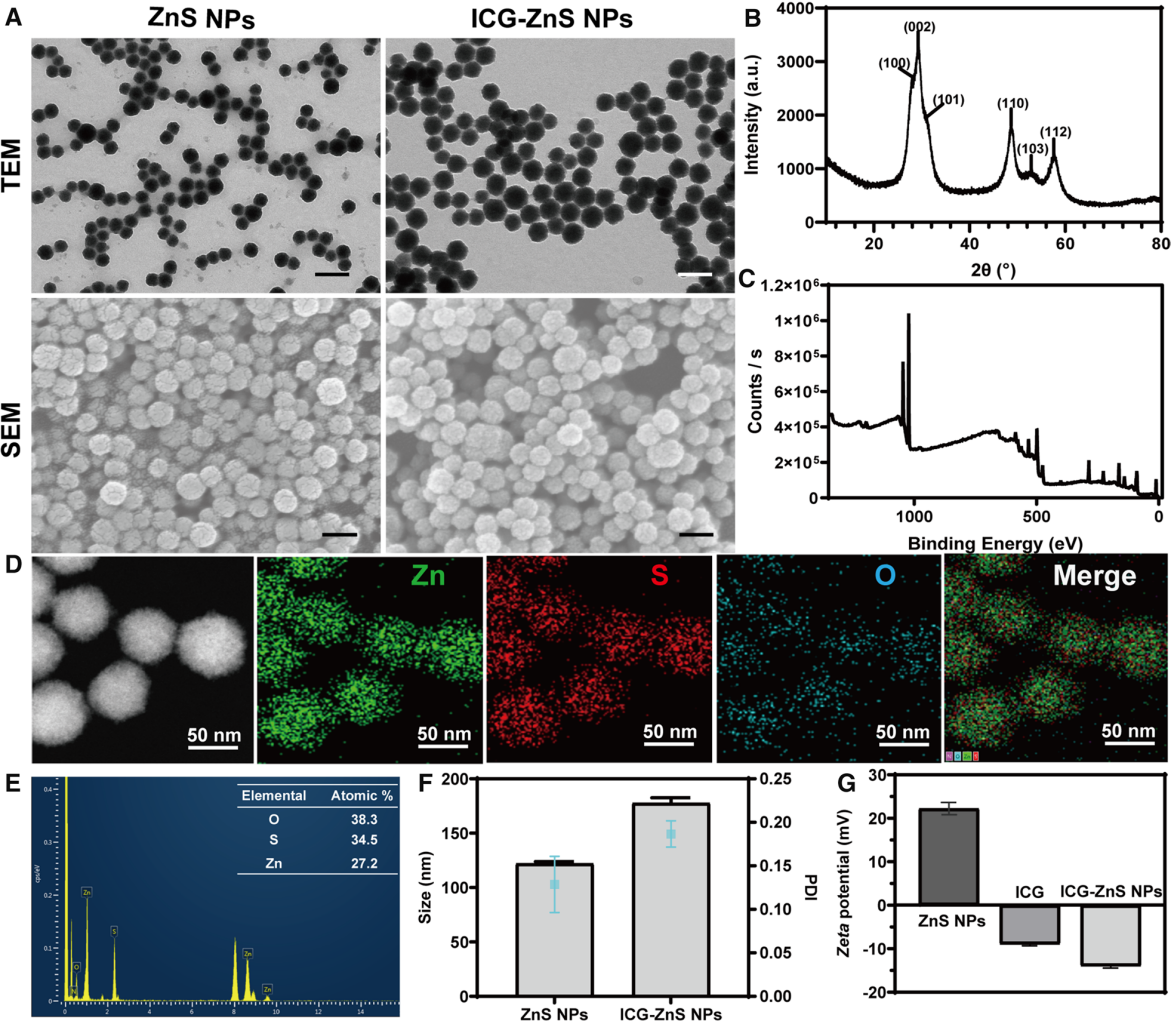
Characterization of ZnS NPs and ICG-ZnS NPs. **A** Representative TEM and SEM images of ZnS NPs and ICG-ZnS NPs (scale bar: 100 nm). **B** XRD patterns of ICG-ZnS NPs. **C** XPS patterns of ICG-ZnS NPs. **D** Elemental mapping of ICG-ZnS NPs (scale bar: 50 nm). **E** EDS survey scanning of the atomic ratios of O, S and Zn in ICG-ZnS NPs. **F** Hydrodynamic size distribution of the various nanoparticles measured by DLS. **G** *Zeta* potential of ZnS NPs, ICG and ICG-ZnS NPs

**Fig. 3:**
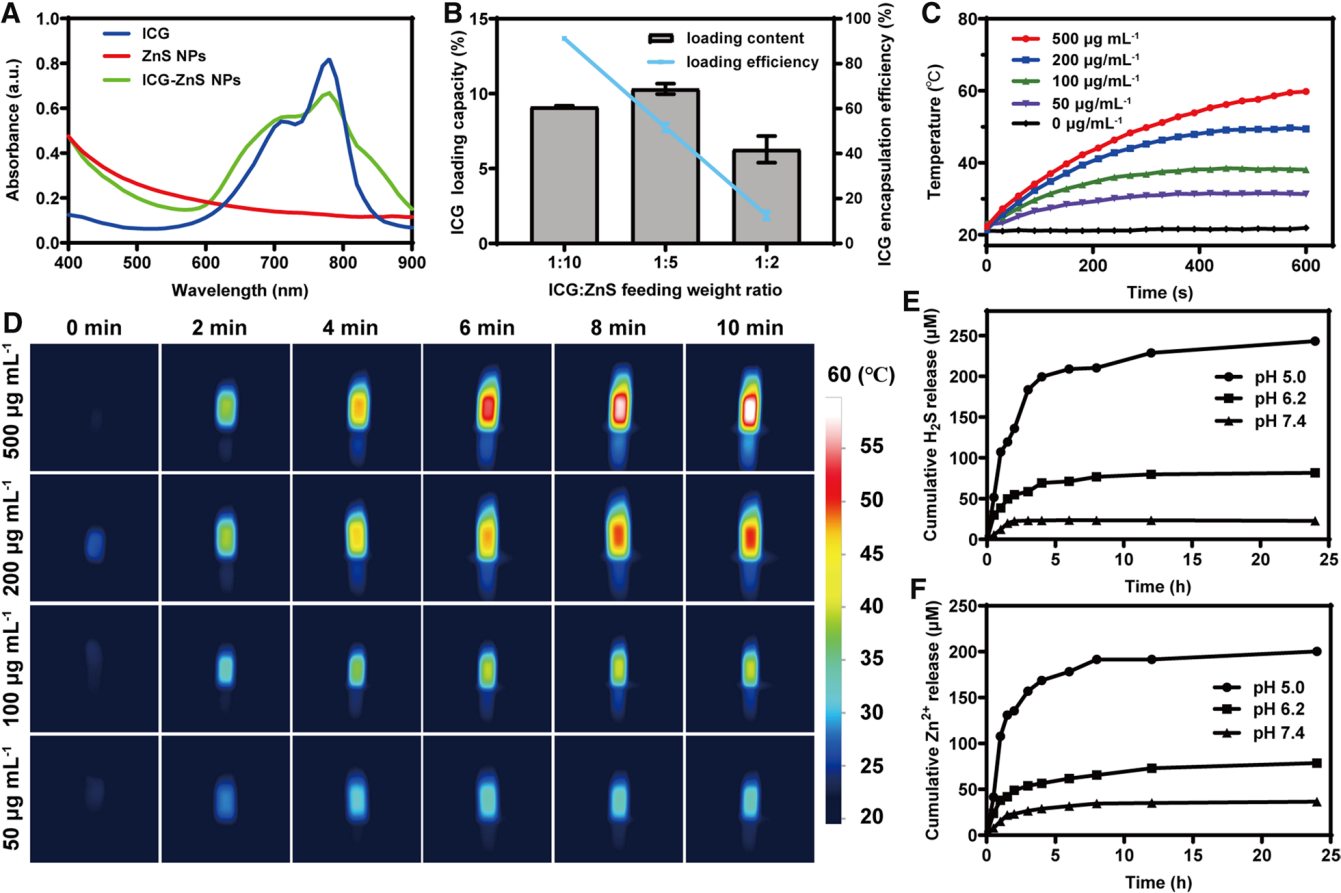
**A** UV–vis absorption of ZnS NPs, ICG and ICG-ZnS NPs. **B** ICG loading capacity and encapsulation efficiency of ICG-ZnS NPs in different mass ratio of ICG/ZnS NPs. **C**, **D** Temperature profile of different concentrations of ICG-ZnS NPs (1 W·cm^−2^). **E** H_2_S release performance of ICG-ZnS NPs with different pH. **F** Zn^2+^ release performance of ICG-ZnS NPs with different pH

ICG-ZnS NPs were synthesized via a two-step procedure. First, ZnS NPs were prepared via the hydrothermal method, according to a previously published protocol [39]. Then, in virtue of the positive surface charge of ZnS NPs, ICG was successfully loaded onto the ZnS NPs surface via electrostatic interaction, to afford the hierarchical structure, generating ICG-ZnS NPs. TEM and SEM observations revealed that ZnS NPs had a well-defined spherical morphology with favorable dispersity and a smooth surface (Fig. 2A). The result of XRD showed six peaks at 27.9°, 29.3°, 31.0°, 48.7°, 53.0°, and 57.8°, corresponding to coordinates (100), (002), (101), (110), (103), and (112) of ZnS (ICSD 9013420), respectively (Fig. 2B). The XRD patterns indicated that the diffraction peaks of coordinates (100) and (101) overlapped with the diffraction peak of coordinate (002) due to the small crystallite size. X-ray photoelectron spectroscopy (XPS) analysis further confirmed the successful preparation of ICG-ZnS NPs, as evidenced by two peaks appearing at approximately 1021.38 and 1044.38 eV that corresponded to Zn 2p and one peak at approximately 161.58 eV that corresponded to S 2p (Fig. 2C and Additional file 1: Fig. S1). From the elemental mapping images (Fig. 2D), the uniform distribution of Zn and S was visualized, demonstrating the successful preparation of ZnS NPs. Energy dispersive spectrometer (EDS) further revealed that the atomic ratios of Zn, S, and O in ICG-ZnS NPs were 27.2%, 34.5%, and 38.3%, respectively (Fig. 2E). ICG loading scarcely altered the intrinsic morphology of bare ZnS NPs, but numerous satellites were found distributed on the surface. Dynamic light scattering was conducted to determine the average particle size and zeta potential. As presented in Fig. 2F and Additional file 1: Fig. S2, after ICG loading, the hydrodynamic diameter increased from 122.13 ± 1.76 nm (PDI 0.13 ± 0.03) of ZnS NPs to 177.73 ± 4.83 nm (PDI 0.19 ± 0.02) of the as-prepared ICG-ZnS NPs. Meanwhile, the zeta potential sharply reversed from 22.23 ± 1.42 mV to -14.00 ± 0.44 mV (Fig. 2G and Additional file 1: Fig. S3), indicating the efficient loading of ICG, which was also confirmed via UV–Vis spectrometer by the appearance of a typical absorbance peak at 780 nm (Fig. 3A). Such a result was likely due to negative charge and amphiphilicity of ICG.

### Evaluation of loading capacity and encapsulation efficiency

To select the optimal ICG loading condition, a series of weight ratios of ICG/ZnS NPs were tested using an enzyme-labeled instrument. As a result, the optimal ICG loading capacity and encapsulation efficiency was achieved, which were 9.21 ± 0.07% and 91.21 ± 0.65%, respectively at the weight ratio of ICG/ZnS NPs = 1:10 (Fig. 3B).

### Photothermal performance of ICG-ZnS NPs

The photothermal capability of nanoparticles was then assessed using an infrared thermography. As displayed in Fig. 3C and 3D, ICG-ZnS NPs demonstrated a dose-dependent temperature increase under laser irradiation (1.0 W cm^−2^), which promptly increased from 22.3 °C to 59.8 °C at the concentration of 500 μg mL^−1^, and increased from 21.4 °C to 49.4 °C at the concentration of 200 μg mL^−1^, whereas no dramatic fluctuation was witnessed for PBS. It is noteworthy that a moderate HT temperature below 50 °C does not cause significant thermal damage to skin tissues [25]. Therefore, NIR laser of 1.0 W·cm^−2^ and a concentration of 200 µg mL^−1^ was selected for subsequent experiments.

### Characterization of H_***2***_S and Zn^2+^ generation

In general, ZnS NPs were nontoxic and biocompatible [40]. It remained stable in neutral water or aqueous sodium hydroxide, environment but decomposed quickly in acid solution, enabling a sensitive responsiveness to BME [33]. Particularly, ZnS NPs continuously released H_2_S and Zn^2+^ in response to acidic conditions of pH 5.0–6.5. In this study, the pH-responsive release behavior of H_2_S and Zn^2+^ from ICG-ZnS NPs was demonstrated at different pH conditions in PBS (pH 7.4, 6.2, and 5.0). As shown in Fig. 3E and F, H2S and Zn^2+^ release was barely detected under neutral conditions. In contrast, the 24 h cumulative H_2_S release at pH = 5.0 and 6.2 was approximately ~ 243 μM and ~ 82 μM, respectively. Additionally, Zn^2+^ release was approximately ~ 200 μM and ~ 78 μM, respectively, demonstrating that ICG-ZnS NPs had effective pH-responsive on-demand delivery of H_2_S gas and Zn^2+^. The pH-responsive H_2_S and Zn^2+^ release was attributed to the acid-triggered decomposition of ICG-ZnS NPs, providing sustainable H_2_S and Zn^2+^ release in the BME.

### Quantification of nanoparticle uptake

**Fig. 4:**
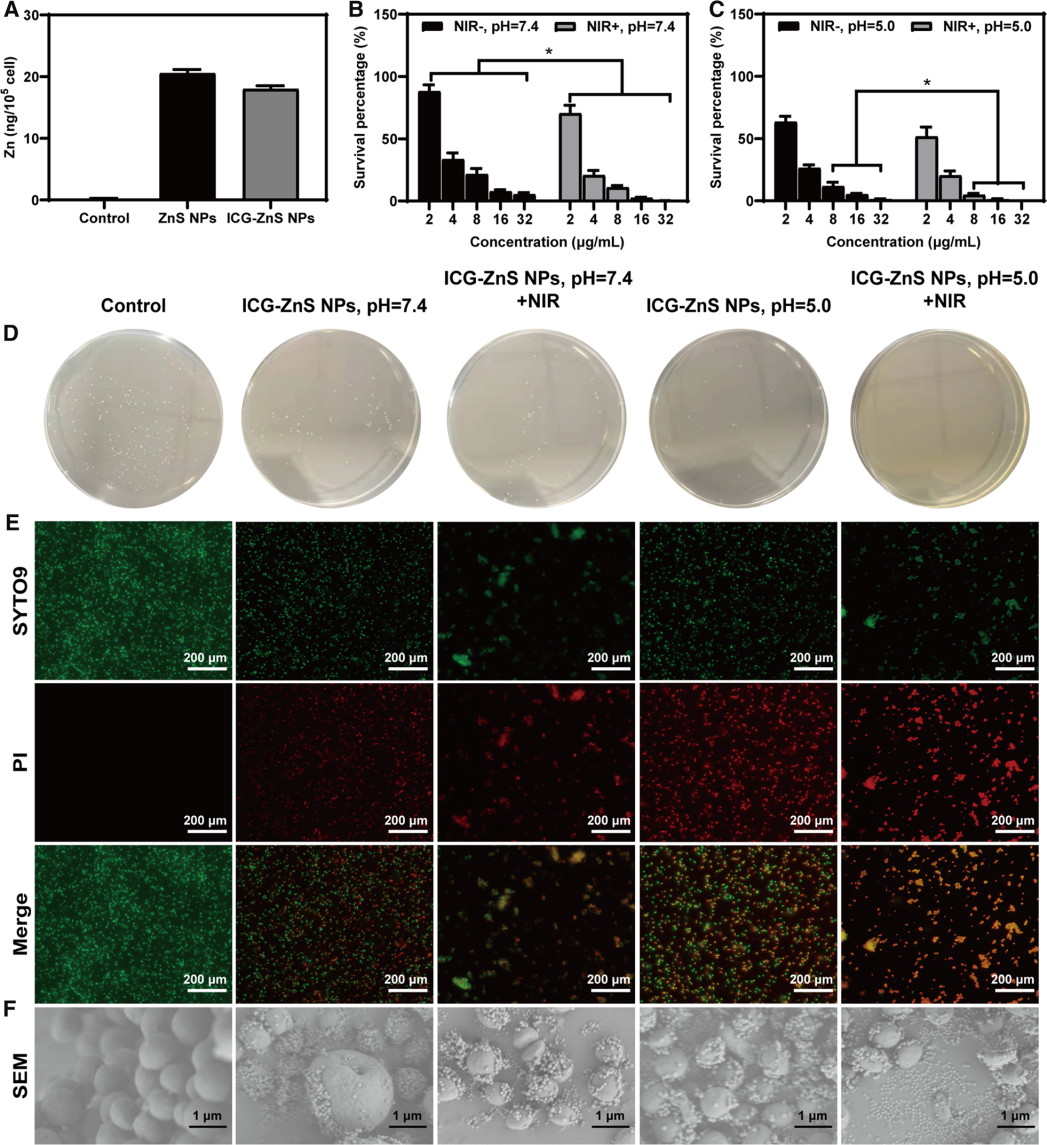
In vitro evaluation of anti-bacterial activity. **A** Intracellular Zn amounts were determined by ICP-MS. **B**, **C** Bacterial survival efficiency treated by the ICG-ZnS NPs and ICG-ZnS NPs + NIR at different concentrations at pH 7.4 and 5.0. **D** Representative plates of MRSA colonies after treatment with ICG-ZnS NPs and ICG-ZnS NPs + NIR (8 µg·mL^−1^) at different concentrations at pH 7.4 and 5.0 (scale bar: 200 μm). **E** Live/dead staining images of MRSA. **F** SEM images of MRSA after different treatments (scale bar: 1 μm). Data are presented as the means ± SD (n = 3), *P < 0.05, **P < 0.01

The quantitative uptake of ZnS NPs and ICG-ZnS NPs by the MRSA was determined with ICP-MS. As shown in Fig. 4A, the content of Zn in the cell is 20.52 ± 0.63 ng (10^5^ cells)^−1^ in ZnS NPs group and is 17.96 ± 0.52 ng (10^5^ cells)^−1^. The results showed no statistically significant between the two groups, indicating that MRSA did not distinguish the uptake of nanoparticles based on the surface charge of the nanoparticles.

### In vitro antibacterial activity of ZnS NPs

Inspired by the superior characteristics of ICG-ZnS NPs, the in vitro antibacterial activity of ICG-ZnS NPs was investigated in different pH conditions using the colony-counting method. Briefly, MRSA was co-cultured with ICG-ZnS NPs at different concentrations (pH = 7.4 or 5.0) before irradiated with NIR laser at 1.0 W cm^−2^ for 10 min, using PBS-treated MRSA as a negative control. As shown in Fig. 4B and C, ICG-ZnS NPs exhibited a concentration-dependent inhibitory effect against MRSA at both pH conditions. At the concentration of 8 μg mL^−1^, ICG-ZnS NPs exhibited an inhibition efficacy of 78.49 ± 4.61% and 88.33 ± 3.20% at pH = 7.4 or 5.0, respectively (*P* < 0.05). With the coupling of NIR laser irradiation, the inhibition efficacy was further enhanced to 89.18 ± 1.63% and 95.26 ± 1.24% (*P* < 0.05), indicating a synergized-antibacterial capability mediated by the hyperthermia effect. Furthermore, under laser irradiation, the inhibition efficacy was further enhanced to 98.77 ± 0.39% and 99.994 ± 0.0014% at both pHs by increasing the ICG-ZnS NPs concentration to 32 μg mL^−1^, fully meeting the clinical requirement. To further investigate the inhibitory effect mediated by temperature, MRSA were treated with ICG + NIR of various concentrations corresponding to different temperature (40 °C, 45 °C and 50 °C). As shown in Additional file 1: Fig. S6, the bacterial survival rate at 45 °C was 4.07 ± 0.48%, indicating that hyperthermia therapy (45 °C) could effectively inhibit the proliferation of MRSA while is difficult to completely eradicate MRSA, therefore combinative participation of ZnS NPs is necessary. The antibacterial activities of all groups were also confirmed by a live/dead staining experiment (Fig. 4E). Consistent with the colony-counting results (Fig. 4D), ICG-ZnS NPs (pH = 5.0 + NIR) demonstrated the most pronounced antibacterial activity, as evidenced by the total overlap of green and red fluorescence, whereas only a part of MRSA was stained with PI after treatment with ICG-ZnS NPs at both pH conditions. SEM images verified the potential eradicating mechanism of each condition: ICG-ZnS NPs displayed preferential adherence to MRSA. Compared to the smooth intact membrane integrity of MRSA in the control group, MRSA in the ICG-ZnS NP group tended to aggregate and membrane deformation and shrinkage were observed, which was further enhanced with NIR irradiation (Fig. 4F). The above results indicated that ICG-ZnS NPs coupled with NIR laser irradiation might provide an antibacterial efficacy higher than that of ICG-ZnS NPs alone in vitro, further demonstrating the synergistic antibacterial therapeutic efficiency of ICG-ZnS NPs.

### In vitro deep biofilm penetration

**Fig. 5:**
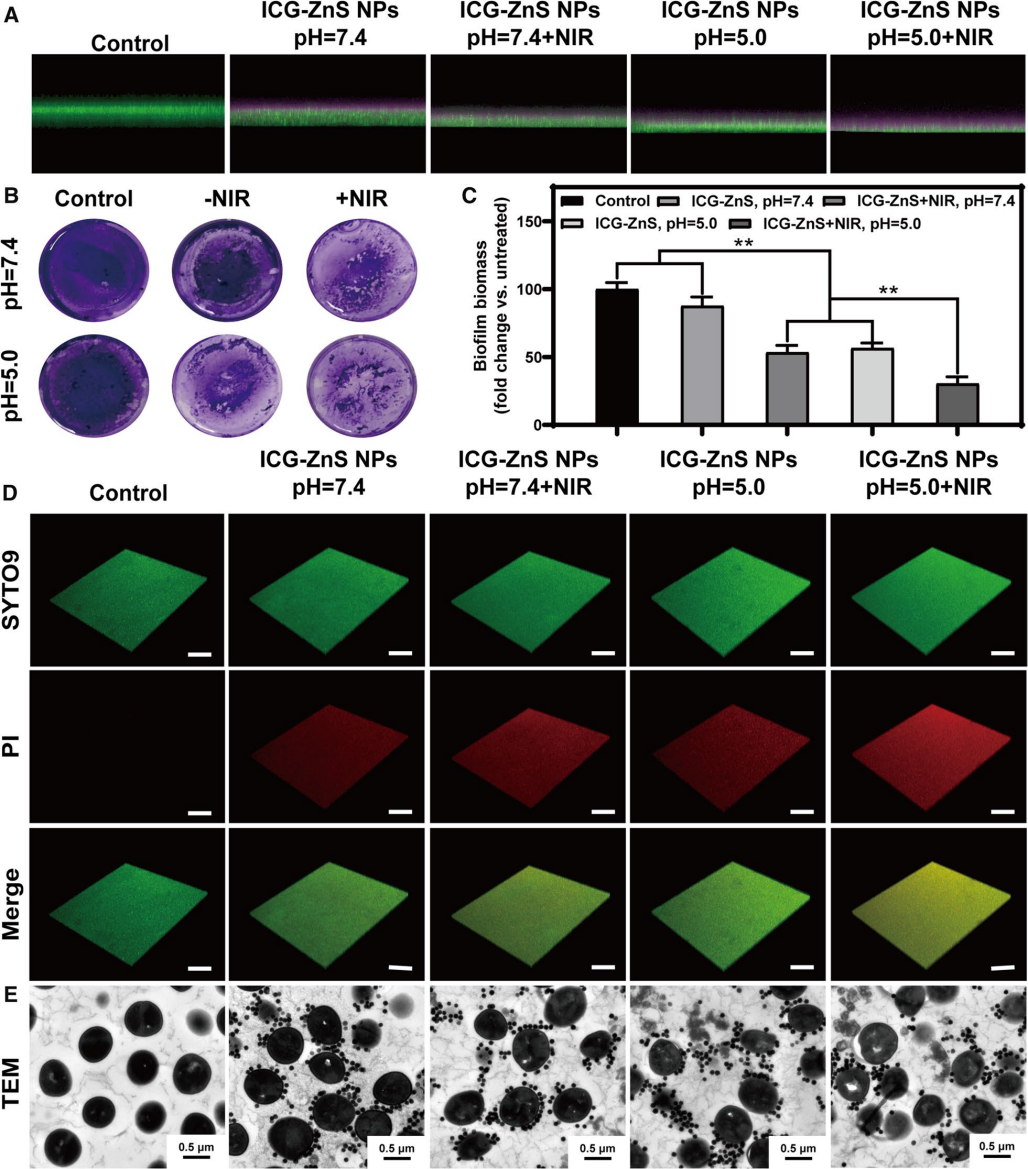
In vitro penetration assay anti-biofilm activity toward MRSA biofilms. **A** CLSM images of MRSA biofilms after treated with ICG-ZnS NPs and ICG-ZnS NPs + NIR at different concentrations at pH 7.4 and 5.0. The presence of biofilm was detected with SYTO 9 dye (green channel), and the nanoparticles were detected with ICG (pink channel). **B** Crystal violet assay to assess the anti-biofilm activity of the samples against an MRSA biofilm. **C** Quantitative analysis of the crystal violet-stained biofilms in panel a by measuring the absorbance at 590 nm. **D** Live/dead staining images of MRSA biofilm using 3D CLSM (scale bar: 100 μm). **E** Typical TEM images of MRSA biofilm treated with different groups (scale bar: 0.5 μm). Data are presented as the means ± SD (n = 3), *P < 0.05, **P < 0.01

A critical prerequisite for biofilm ablation is whether the NPs efficiently penetrate the dense protective layers of biofilm [41]. To evaluate the penetration capability of ICG-ZnS NPs toward the interior of the MRSA biofilms, the MRSA biofilms was incubated with ICG-ZnS NPs for 120 min at pH 7.4 or 5.0, followed by subjection to CLSM and a z-stack image. For visualization of the whole biofilms, SYTO 9 was used to stain all bacteria in the MRSA biofilms, noted in green, while the ICG fluorescence was pink. As shown in Fig. 5A, ICG-ZnS NPs treatment at pH 7.4 without NIR irradiation showed limited penetration potency, as observed from weak fluorescence of ICG on the biofilm surface. After incubation at pH 5.0 and NIR irradiation, significantly enhanced penetration potency was achieved, reflected by deep-diffused fluorescence of ICG within the biofilms. These results indicated that the H_2_S release in BME and the NIR irradiation-mediated hyperthermia effect potentiated penetration into the MRSA biofilms.

### In vitro anti-biofilm activity of ICG-ZnS NPs

The anti-biofilm activity of ICG-ZnS NPs against MRSA biofilms was examined using crystal violet staining method and live/dead staining assay. MRSA biofilms were incubated with ICG-ZnS NPs (200 μg mL^−1^) in different pH conditions with or without laser irradiation. As shown in Fig. 5B, crystal violet staining indicated that biofilms treated with ICG-ZnS NPs + NIR exhibited a distinctly reduced amount of crystal violet stainable biomass in comparison with other groups (*P* < 0.01). For quantitative analysis of the anti-biofilm effect of ICG-ZnS NPs, the absorbance after treatment was detected using a UV–Vis spectrometer (Fig. 5C). The result of live/dead staining assay further illustrated the anti-biofilm activity of ICG-ZnS NPs in vitro (Fig. 5D). The ICG-ZnS NPs at pH 5.0 combined with NIR irradiation displayed strong red fluorescence over the whole biofilm, confirming significant bacterial killing and biofilm clearance and demonstrating the highly effective bactericidal performance for formed biofilms at pH 5.0. Afterward, TEM observation was conducted to visualize the membrane structure of MRSA treated with different group (Fig. 5E). After treatment with PBS, MRSA maintained original spherical shape and membrane structure. Treatment with ICG-ZnS NPs at pH 7.4 led to membrane destruction with cracked bilayer structure in a small quantity of MRSA. Meanwhile, the increased rupture of MRSA bacteria can be observed when treated with NIR irradiation or at pH 5.0. In addition, the group treated with NIR irradiation at pH 5.0 resulted in the complete rupture of the membranes with severe matrix outflow. Taken together, ICG-ZnS NPs coupled with NIR irradiation possessed remarkable biofilm penetration and photothermal eradication of MRSA biofilms.

### In vitro anti-inflammation evaluation

**Fig. 6:**
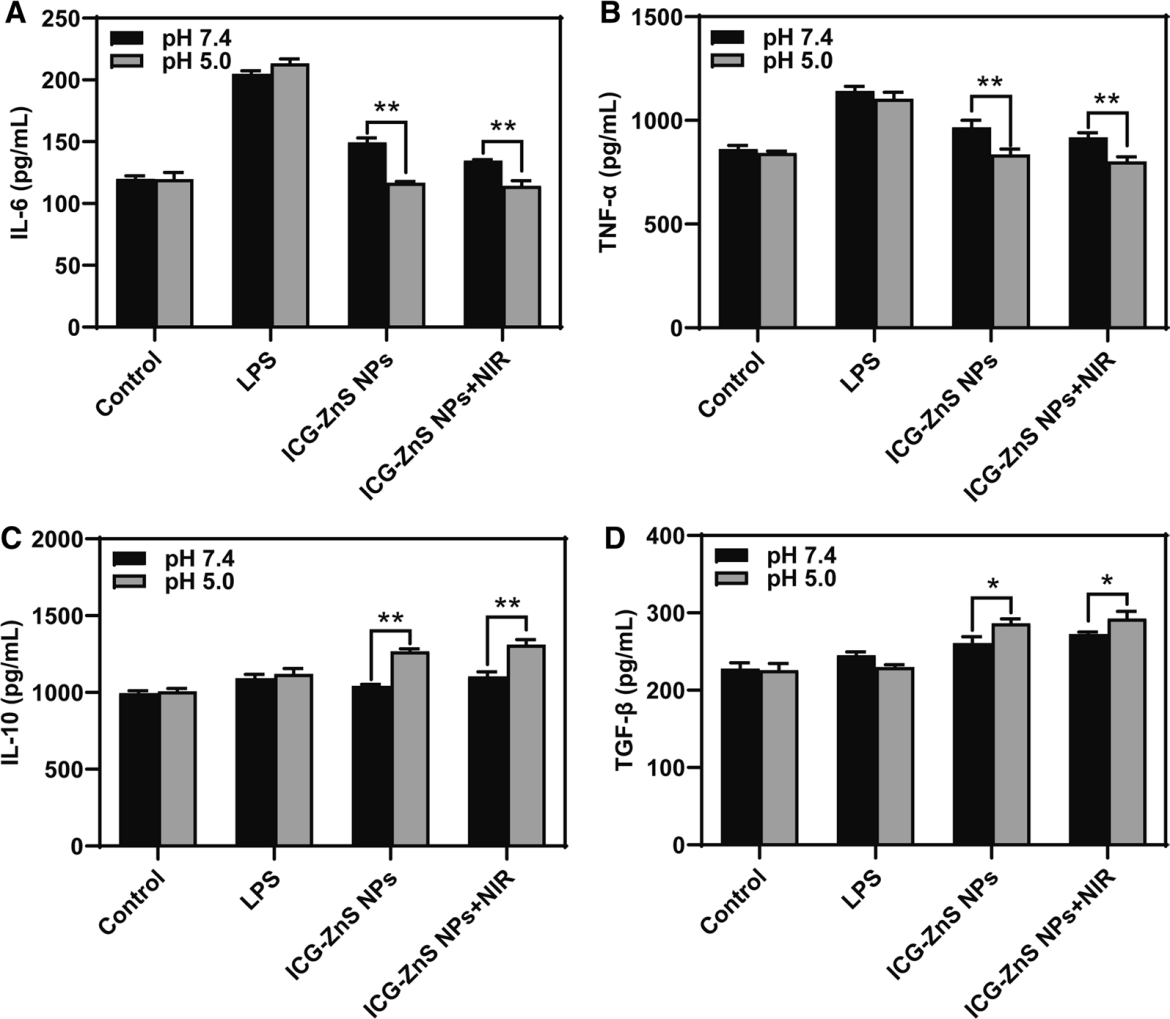
**A**, **B** ELISA assays of typical proinflammatory cytokines, IL-6 and TNF-α, in macrophages after different treatments (200 µg mL^−1^), respectively. **C**, **D** ELISA assays of typical anti-inflammatory cytokines, IL-10 and TGF-β, in macrophages after different treatments, respectively. Data are presented as the means ± SD (n = 3), *P < 0.05, **P < 0.01

To evaluate the potential anti-inflammatory capability of ICG-ZnS NPs, macrophages were used as representative inflammatory cells in vitro. As shown in Fig. 6A and 6B, when treated with ICG-ZnS NPs, the contents of proinflammatory cytokines including IL-6 and TNF-α decreased compared with that of the LPS-treated group, which are mainly ascribed to the release of H_2_S from ZnS NPs. Interestingly, the proinflammatory cytokines were further decreased significantly at pH 5.0 compared with condition of pH 7.4 (*P* < 0.01) and are comparable to the control group due to more H_2_S releases. As shown in Fig. 6C and D, ICG-ZnS NPs at pH 5.0 displayed a much higher level of IL-10 than other groups (*P* < 0.01). Similarly, the TGF-β levels in pH 5.0 groups were also significantly higher than those at pH 7.4 (*P* < 0.05). Taken together, the above results showed the potential anti-inflammatory ability of the ICG-ZnS NPs, which can accelerate the healing process.

### In vivo anti-biofilm activity and wound healing evaluation

**Fig. 7:**
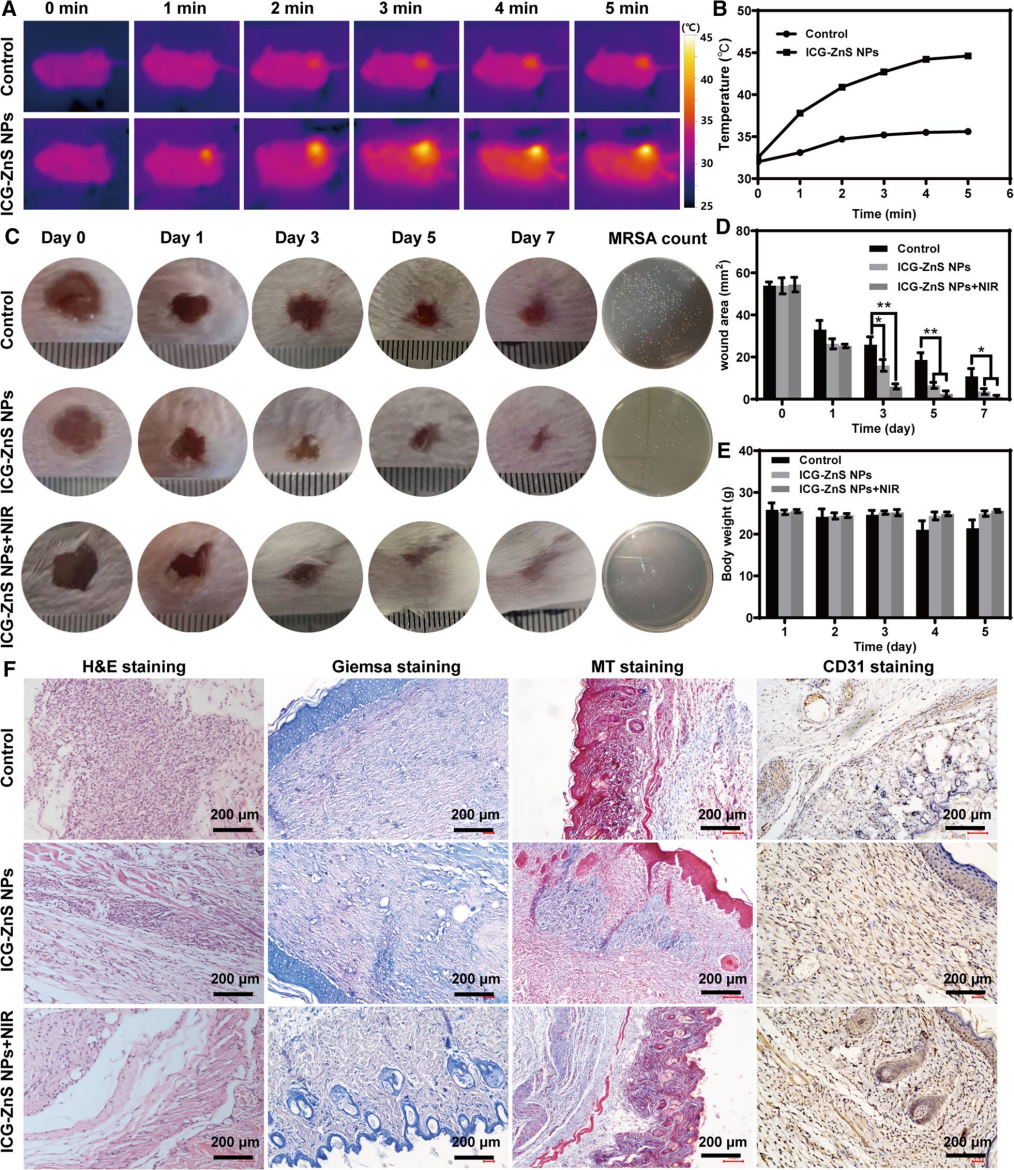
In vivo anti-biofilm activity and wound healing evaluation. **A**, **B** Representative real-time thermal images of the mice treated with PBS and ICG-ZnS NPs irradiated with NIR (808 nm, 1 W cm^−2^). **C** Wound photograph of MRSA infected mice after treated with PBS, ICG-ZnS NPs and ICG-ZnS NPs + NIR for 0–7 days; Quantitative measurement of bacteria counts in wounds by standard plate count. **D** Quantitative analysis of relative wounds area over time. **E** Body weight changes during 7 days. **F** H&E staining images, Giemsa staining images, MT staining images and CD31 immunostaining images of skin tissues after treatments with PBS, ICG-ZnS NPs and ICG-ZnS NPs + NIR (scale bar: 200 μm). Data are presented as the means ± SD (n = 3), *P < 0.05, **P < 0.01

A chronic wound model with severe infection was established to further assess the anti-biofilm efficacy of ICG-ZnS NPs in vivo. Briefly, to form an initial abscess, a small 7 mm diameter wound was infected with MRSA (200 μL, 10^8^ CFU mL^−1^) for 24 h. After treatment with ICG-ZnS NPs, the temperature variation trend was evaluated under the stimulation of NIR irradiation. As shown in Fig. 7A and B, the temperature at ICG-ZnS NPs treated-wounds rapidly increased from 32.5 to 44.6 °C after irradiated with NIR laser for 5 min. In contrast, only unnoticeable temperature change could be detected for the control group. As shown in Fig. 7C and D, the suppurative symptom and inflammatory resonation at the wound treated with PBS reveals that wounds with biofilm formation have low levels of self-healing. However, the wounds treated with ICG-ZnS NPs + NIR presented benign phenomena, including wound healing and complete disappearance of the wound. Similarly, the bodyweight of mice steadily increased after treatment with ICG-ZnS NPs + NIR (Fig. 7E). There was no viable MRSA in the ICG-ZnS NPs + NIR treated abscess after incision, whereas using quantitative analysis, there was a large amount of viable MRSA at wound treated with PBS.

Furthermore, histological analysis was conducted to evaluate the anti-biofilm efficacy. We evaluated inflammation, collagen deposition, bacteria count and angiogenesis by H&E staining, MT staining, Giemsa staining and CD31 immunohistochemistry staining, respectively (Fig. 7F). H&E staining results showed significantly attenuated degree of neutrophil infiltration in the ICG-ZnS NPs + NIR group. Results of Giemsa-staining further indicated that ICG-ZnS NPs + NIR can effectively kill bacteria in infected wound. Additionally, Masson’s trichrome staining and CD31 staining photographs showed accelerated collagen deposition and new blood vessel formation at infected wound after treatment with ICG-ZnS NPs + NIR. These results suggested that ICG-ZnS NPs combined with hyperthermia therapy had a satisfactory anti-biofilm and wound-healing performance at the abscess site.

### In vivo anti-inflammation evaluation

**Fig. 8:**
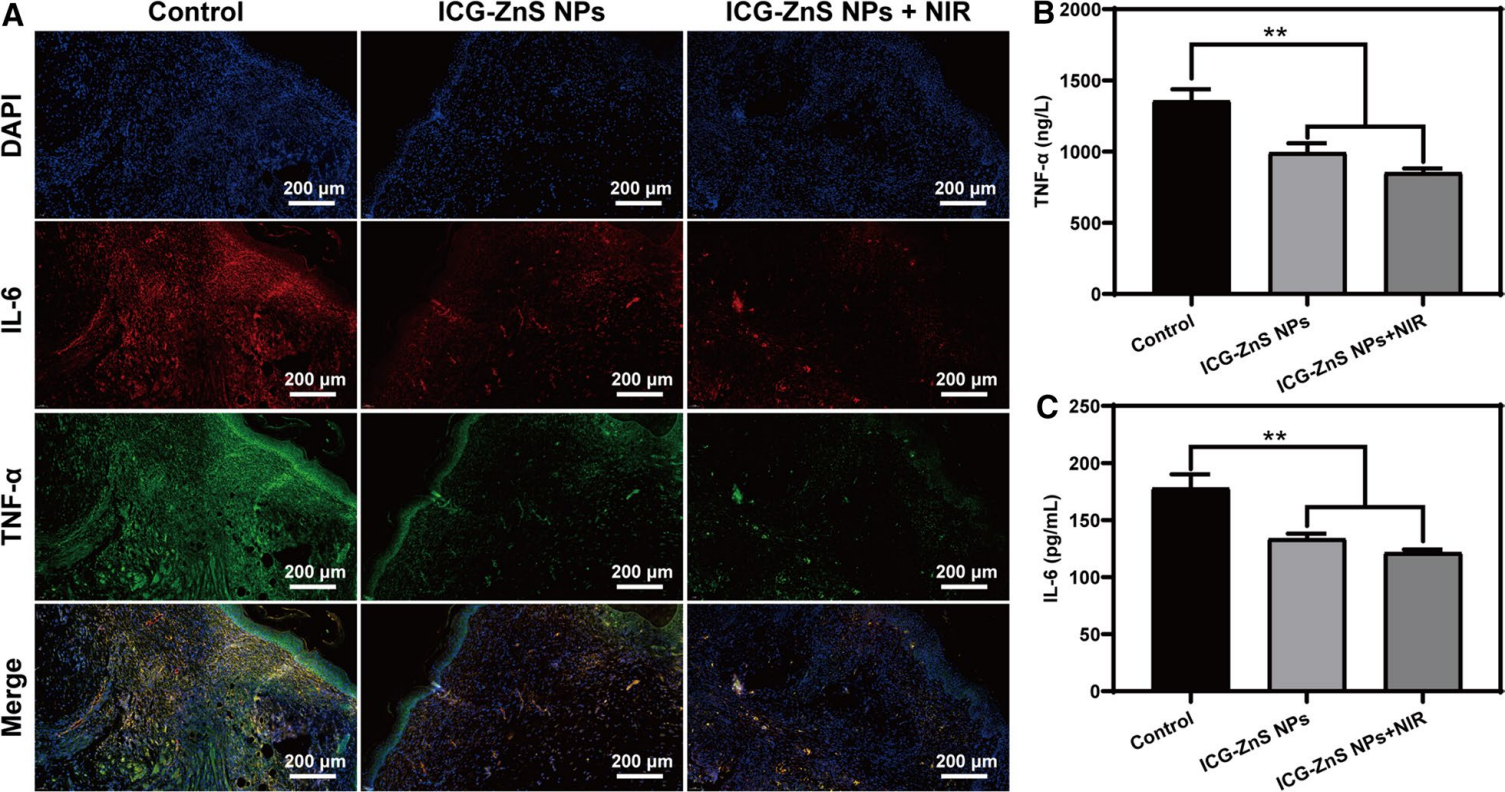
In vivo anti-inflammation evaluation. **A** Histological analysis of tissue sections through TNF-α and IL-6 immunofluorescence staining on day 7 after various treatments (scale bar: 200 μm). **B**, **C** ELISA assays of TNF-α and IL-6 after various treatments. Data are presented as the means ± SD (n = 3), *P < 0.05, **P < 0.01

H_2_S has demonstrated considerable therapeutic potential in clinical treatment of various inflammatory diseases by suppressing inflammatory responses [42]. To assess the anti-inflammatory ability of ICG-ZnS NPs, double immunofluorescent staining and a corresponding ELISA kit were used to quantitatively analyze the typical proinflammatory cytokines including TNF-α and IL-6. The images of control group showed an abundance of red and green fluorescent spots indicated the large amounts of inflammatory factors are secreted (Fig. 8A). In comparison to control groups, the secretion of inflammatory factors was reduced and inflammation was weakened when infected wounds received ICG-ZnS NPs + NIR treatment. Interestingly, although without NIR irradiation, compared with the control group, the amounts of TNF-α and IL-6 secretion in wounds treated with ICG-ZnS NPs was reduced significantly, which was mainly ascribed to the effective anti-inflammatory effect of H_2_S release from ICG-ZnS NPs. In addition, ELISA analysis was comparable to TNF-α and IL-6 immunofluorescent staining; Treatment of ICG-ZnS NPs + NIR considerably decreased the expression of TNF-α and IL-6 compared to those of the control groups (*P* < 0.01) (Fig. 8B and C). Collectively, these results indicated that ICG-ZnS NPs retained their anti-inflammatory function in vivo.

### Biosafety study of ICG-ZnS NPs

**Fig. 9:**
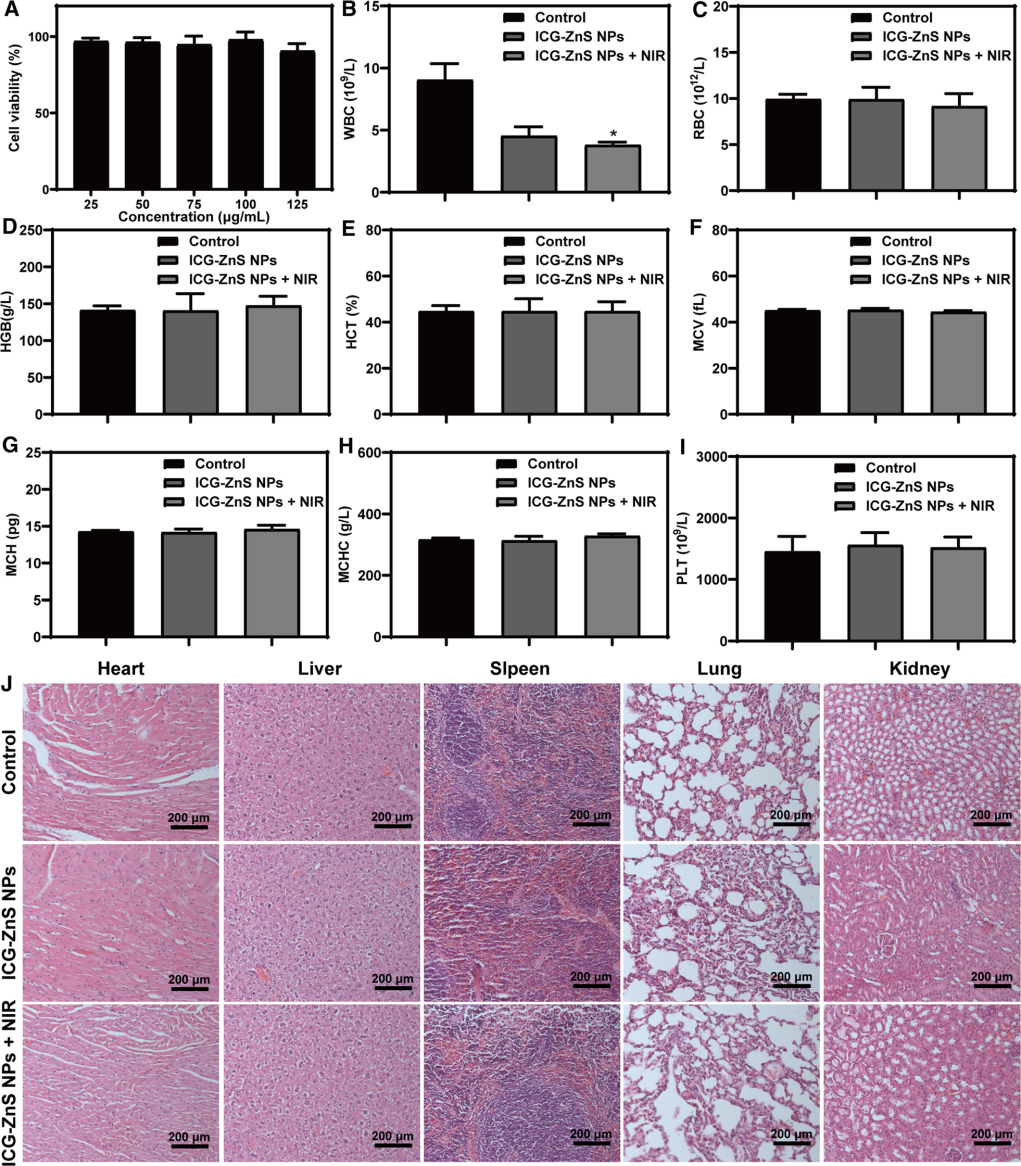
In vivo biosafety study of ICG-ZnS NPs. **A** Cell viability of at different concentrations of ICG-ZnS NPs. **B**–**I** The blood routine test on day 7 of PBS, ICG-ZnS NPs and ICG-ZnS NPs + NIR groups. **J** H&E staining of heart, liver, spleen, lung and kidney after treated with PBS, ICG-ZnS NPs and ICG-ZnS NPs + NIR (scale bar: 200 μm). Data are presented as the means ± SD (n = 3), *P < 0.05

The biosafety of ICG-ZnS NPs + NIR is critical for its further clinical translation. First, the cytocompatibility of ICG-ZnS NPs was evaluated in vitro via a MTT assay by incubating ICG-ZnS NPs with NIH-3T3 fibroblasts for 24 h. When cells were treated with ICG-ZnS NPs at concentrations of 25–125 µg·mL^−1^, the cell viabilities were all above 90%, respectively (Fig. 9A). These data suggested that ICG-ZnS NPs were less toxic to normal cells within the therapeutic concentration. To evaluate the biosafety of ICG-ZnS NPs in vivo, Blood routine analysis showed that compared with the control group, the white blood cell levels of the ICG-ZnS NPs and ICG-ZnS NPs + NIR groups decreased (*P* < 0.01) (Fig. 9B), which may be related to wound healing and inflammation elimination, while other blood routine indexes were not significantly abnormal (Fig. 9C–I). H&E staining of major organs and blood biochemistry analysis were performed. H&E staining images of heart, liver, spleen, lung, and kidney showed no pathological sign in major organs, further indicating the excellent biosafety of ICG-ZnS NPs (Fig. 9J).

## Conclusion

In summary, the study developed a biofilm-responsive hierarchical H_2_S-releasing ICG-ZnS NPs, which has a dual function of concomitantly deep-penetrating MRSA and alleviating inflammatory responses in MRSA infected wounds. The inhibitory effect on as-constructed biofilms were arising from the synergistic effects of Zn^2+^, H_2_S-releasing and HT. Concretely, H_2_S-releasing could not only promote ICG-ZnS NPs penetration, but also effectively ameliorate the inflammatory responses by. In addition, Zn^2+^, H_2_S gas and HT jointly accelerated the MRSA death. Due to these advantageous features, ICG-ZnS NPs with NIR-irradiated accelerated wound healing. Overall, the reported biofilm-responsive hierarchical H_2_S-releasing ICG-ZnS NPs presented a feasible effective and biosafe approach for treatment of MRSA biofilm-infected wounds in clinic.

## Methods

### Materials

Polyvinylpyrrolidone (PVP, K30) were acquired from Sigma Co. Ltd. Indocyanine green (ICG), zinc acetate, thiourea and iron (III) chloride (FeCl_3_) were obtained from Aladdin Co. Ltd. 3-(4,5-Dimethylthiazol-2-yl)-2,5-diphenyltetrazolium bromide (MTT) and *N*,*N*-Dimethyl-*p*-phenylenediamine dihydrochloride (DMPD) were obtained from Yuanye Biological Technology Co. Ltd. Tryptic soy broth (TSB) medium were acquired from Solarbio Science & Technology Co., Ltd. Sodium sulfide and crystal violet were purchased from Macklin Co. Ltd. The LIVE/DEAD BacLight Bacterial Viability Kit was bought from Thermo Fisher Scientific, Inc. All antibody including anti-CD31, anti-IL-6 and anti-TNF-α were purchased from Affinity Biosciences. Mouse enzyme linked immunosorbent assay (ELISA) kits including TNF-α and IL-6 were purchased from Jiangsu Meimian industrial Co., Ltd. Deionized (DI) water was purified by PALL Life Sciences ultrapure water system.

Fibroblast cells (NIH 3T3) were obtained from the cell bank of ATCC. Fetal bovine serum (FBS), penicillin G sodium, streptomycin sulfate and DMEM medium were acquired from Gibco BRL.

### Preparation of ZnS NPs

ZnS NPs were prepared using a modified hydrothermal method, as previously reported [39]. Briefly, 2 mmol zinc acetate was added to 50 mL DI water. Then, 2 g PVP (K30) were added and dispersed evenly with a vortex (5 min, 1200 rpm). Subsequently, 40 mmol thiourea were added and the mixture was stirred for 20 min at 25 °C. The mixture was then moved to a 100 mL autoclave with an inner polytetrafluoroethylene lining and kept at 140 °C for 1 h. After cool down for 4 h at room temperature, the prepared precipitant was collected by centrifugation and washed with water three times (13,000 rpm, 10 min).

### Evaluation of loading capacity and encapsulation efficiency

ICG-ZnS NPs were prepared using the electrostatic adsorption method. Briefly, different masses of ICG were mixed with 1 mg ZnS NPs in 10 mL DI water at different w/w ratios (ICG: ZnS NPs = 1:10, 2:10, and 5:10). Then, the solution was constantly stirred at dark for 24 h, in order to promote ICG loaded into ZnS NPs completely by electrostatic attraction. The yielded ICG loaded ZnS NPs (ICG-ZnS NPs) were collected via centrifugation (13,000 rpm, 10 min) and the residual ICG and washing water were collected. An ultraviolet–visible spectrophotometer was used to determine the unloaded ICG content to calculate the drug loading capacity (LC, the percentage of the amount of drug loaded into the nanoparticles to the weight of nanoparticles) and encapsulation efficiency (EE, the amount of drug loaded into the nanoparticles to the total amount of drug) of ICG-ZnS NPs. The drug LC and EE were calculated according to the following formula:

1 $$ {\text{LC }}\left( \% \right) \, = \, \left( {\text{The loaded drug mass}} \right)/\left( {{\text{The loaded drug mass }} + {\text{ The total mass of nanoparticles}}} \right) \, \times { 1}00\% $$

2 $$ {\text{EE }}\left( \% \right) \, = \, \left( {\text{The loaded drug mass}} \right)/\left( {\text{The total drug mass}} \right) \, \times { 1}00\% $$

### Characterization of ZnS NPs and ICG-ZnS NPs

The ICG-ZnS NPs morphology was observed using transmission electron microscopy (TEM, H-7650, Hitachi, Japan) and scanning electron microscopy (SEM, SU8010, Hitachi, Japan). The elemental mapping and energy dispersive X-ray spectroscopy (EDS) spectra of ICG-ZnS NPs were observed using TEM (Tecnai G^2^ F20 S-TWIN, FEI, USA). Powder X-ray diffraction (XRD) patterns were measured via a D/Max-RB X-ray diffractometer (Panalytical X'Pert'3 Powder, Malvern, UK) at a scan rate of 2°/min. Elemental distribution was characterized via X-ray photoelectron spectroscopy (XPS, Thermo Scientific K-Alpha, Thermo, USA). Hydrodynamic size and zeta potential of the nanoparticles were measured via Zetasizer (Nano ZS90, Malvern, UK). The UV–Vis adsorption was characterized through an enzyme-labeled instrument (SpectraMax M2, Molecular Devices, USA).

### Characterization of H_2_S and Zn^2+^ generation

H_2_S generation from ICG-ZnS NPs was measured by methylene blue colorimetry according to the previous research [43]. Briefly, take 1 mL solution mixed with 1 mL Zn(Ac)_2_/Na(Ac) solution (4:1 mass ratio). Subsequently, 0.5 mL DMPD (1.5 mg mL^−1^) and FeCl_3_ (5 mg mL^−1^) were added. After incubation for 20 min, the methylene blue was formed and the absorbance can be examined at 665 nm via enzyme-labeled instrument. In order to quantify the concentration of H_2_S release at each time point, Na_2_S was used to establish a standard curve. Meanwhile, the Zn^2+^ generation from ICG-ZnS NPs was examined via an Inductive Coupled Plasma Emission Spectrometer (ICP, X Series II, Thermo, USA). For H_2_S and Zn^2+^ release properties, 2.50 mg ICG-ZnS NPs were dispersed in 10 mL PBS solution with different pH (5.0, 6.2, and 7.4). Subsequently, the mixtures were placed in a gas bath constant-temperature oscillator at 37 °C. At different time points (0, 0.5, 1, 2, 4, 8, 12, and 24 h), 2 mL supernatant was collected for further measurements by centrifugation (13,000 rpm, 10 min), and then another 2 mL fresh PBS was added.

### Photothermal property study of ICG-ZnS NPs

Four different concentrations of ICG-ZnS NPs (0.05, 0.2, 0.1, and 0.5 mg mL^−1^) were irradiated with an 808 nm NIR laser (Changchun Feimiao Technology Co., Ltd., China) with 1.0 W·cm^−2^ power densities for 10 min. The control group used PBS as a contrast for the same procedure and 1 mL was used for each of the above preparations. A digital thermometer (Testo 872, Testo SE & Co. KGaA, Germany) with a thermocouple probe was applied to record the temperature at each time points of the different solutions.

### Quantification of nanoparticle uptake

Internalization of nanoparticles was determined by a quantitative method based on the Zn content measured by ICP-MS. MRSA were cultured with ZnS NPs and ICG-ZnS NPs for 6 h at 37 °C. Untreated cells were used as controls. Bacterial suspensions were washed three times with PBS. In order to removed liquid media, MRSA were dried by gentle heating. Subsequently, 1 mL of H_2_SO_4_: HNO_3_ (1:9) mixture was added to flasks and kept for 1 day for digestion.

### In vitro evaluation of anti-biofilm activity

Five groups were used: control (PBS), ICG-ZnS NPs (pH = 5.0), ICG-ZnS NPs + NIR (pH = 5.0), ICG-ZnS NPs (pH = 7.4), and ICG-ZnS NPs + NIR (pH = 7.4). Mature MRSA bacteria or biofilms were treated with ICG-ZnS NPs at different pH and then were treated with or without NIR laser irradiation (808 nm, 1.0 W cm^−2^).

### Bacterial plate killing assays

MRSA was employed in all antibacterial experiments. To acquire MRSA, inoculate several individual colonies into TSB medium and then cultured at 37 °C for 16–18 h until stationary phase was reached. After that, the culture solution (40 μL) was diluted with fresh TSB medium (4 mL) and re-grow to mid-log phase (OD_600_ = 0.5) at 37 °C. MRSA were harvested and washed twice with PBS via centrifugation (6000 rpm, 10 min). Before seeded MRSA to the 96-well microplate, the suspension was adjusted to 1.5 × 10^6^ colony forming units (CFUs) mL^−1^ with PBS. The MRSA suspension (50 μL) and different concentrations of ICG-ZnS NPs (100 μL) were co-incubated at different pH for 3 h. Subsequently, ICG-ZnS NPs + NIR (pH = 7.4) and ICG-ZnS NPs + NIR (pH = 5.0) group were irradiated with NIR for 10 min. Then, serial tenfold dilutions were generated using PBS. For resulting in visible colonies, diluted bacterial solutions (20 μL) were plated onto TSB agar plates, and then the plates were incubated at 37 °C overnight. Each trial was performed in triplicate, and the reported results are the average of three independent trials.

### Live/dead bacterial staining

After treatment in different groups, SYTO 9 (2 μM) and propidium iodide (PI, 1 μM) dyes were added to the MRSA suspensions in the dark and incubated for 15 min. Then, removed free SYTO 9 and PI via centrifugation, and the resulting bacterial pellets were washed twice with PBS. To fixed the collected MRSA, 4% paraformaldehyde solution was added. The resultant MRSA suspension was transferred onto a cover-slip, air-dried and immersed with mounting oil. Finally, the images were observed by Upright fluorescence microscope (AXIO SCOPE.A1, Carl Zeiss AG, Germany). SYTO-9 and PI were characterized using fluorescein isothiocyanate and tetramethyl rhodamine filters, respectively. All bacteria stained green, whereas those with compromised cytoplasmic membranes were stained red.

### Evaluation of MRSA by SEM

Bacterial suspensions (at mid log phase) were prepared as mentioned above and treated with ICG-ZnS NPs at different pH or laser conditions. Bacteria obtained by centrifugation were washed twice with PBS and fixed with 2.5% glutaraldehyde at 4 °C overnight. Then, samples were post-fixed in 1% osmic acid for 1 h, and dehydrated with a series of graded ethanol solutions and a tert-butanol series (50%, 75%, 90%, and 100%), each for 10 min, respectively. After drying, the samples were coated with platinum and observed by SEM.

### Bacterial biofilms formation and harvesting

Logarithmic bacteria growing in TSB medium were added to a laser confocal dish (1 × 10^6^ cells well^−1^) and incubated at 37 °C constant temperature incubator for 48 h. During the culture, replace TSB media every 24 h for maintain biofilms nutritional status. Subsequently, the medium was removed, washed with PBS carefully to remove the planktonic bacteria. Finally, the MRSA biofilms were harvested for further experiment.

### Crystal violet staining assay

MRSA biofilms were treated with PBS and ICG-ZnS NPs (0.25 mg mL^−1^) at different pH or laser conditions for 3 h at 37 °C. Subsequently, MRSA biofilms were stained with 0.1% crystal violet for 15 min. After the biofilms were washed carefully with DI water, 33% v/v acetic acid was added to the plate for solubilizing the crystal violet stain. Finally, the absorbance of each dish at 560 nm was measured to assess biofilm biomass.

### In vitro deep biofilm penetration and biofilm live/dead staining

Treated MRSA biofilms were simultaneously stained with SYTO 9 and PI dyes for 30 min in the dark. Then, a confocal laser scanning confocal microscope (CLSM880, Carl Zeiss AG, Germany) was used to image the fluorescence. To observe biofilm accumulation and the penetration capability of the NPs, the fluorescence of ICG was also observed. Images were collected and Z-stacks were compiled into 3D images.

### MRSA membrane structure evaluation using TEM

TEM was performed to observe MRSA membrane structures in biofilms. Briefly, MRSA biofilms incubated with ICG-ZnS NPs (250 μg mL^−1^) at different pH or laser conditions were collected by centrifugation (6000 rpm, 5 min). Then, the MRSA biofilms form different groups were fixed with 2.5% glutaraldehyde solution at 4 °C for more than 4 h. Subsequently, the fixed MRSA biofilms was washed once with PBS. Afterward, 1% osmic acid were added to sample for 1 h and then washed with PBS. Samples were continuously dehydrated in a series of concentrations (50%, 70%, 80%, 90%, and 100%) of ethanol and tert-butanol, respectively.

### In vitro anti-inflammation evaluation

RAW264.7 macrophages with density of 2 × 10^4^ cells well^–1^ were seeded into 48 well-plate and incubated at 37 °C for 6 h. Then, LPS (1 μg mL^–1^) was added and for another 24 h. After which, ICG-ZnS NPs (200 μg mL^–1^) were added and incubated with macrophages at 37 °C for 24 h, and different groups were treated with or without NIR irradiation (1 W cm^–2^, 10 min). Then, the supernatants in different groups were collected. The expression levels of the secretion of inflammatory cytokines and anti-inflammatory cytokines were evaluated by ELISA assay, including IL-6, TNF-α, IL-10 and TGF-β.

### In vitro cytocompatibility evaluation

A cytotoxicity evaluation was performed using the MTT assay. Briefly, NIH 3T3 with density of 1 × 10^4^ cells well^−1^ were seeded into 96-well plates and incubated at 37 °C overnight. Subsequently, cells were treated with serial concentrations (25, 50, 75, 100, and 125 μg mL^−1^) of ICG-ZnS NPs. After incubation for 24 h, culture medium containing 1 mg mL^−1^ MTT was added to each well with a further incubation for 4 h. Then, the medium containing MTT was removed, and 150 μL DMSO was added. Cell viability was determined by recording the absorption values at wavelength of 490 nm with a microplate reader.

### Establishment of a mouse cutaneous wound infection model

BALB/c mice (6–8 weeks old, male, 20–25 g) were purchased from Shanghai SLAC Laboratory Animal Co., Ltd. To create the mouse cutaneous wound infection model, hair was removed on the hind legs with a shaving device. After confirming that the leg skin was not damaged, pentobarbital was injected intraperitoneally according to mouse body weight. After mice were completely anesthetized, a regular round wound was made in the hind leg hair removal area, avoiding the fascia layer. Mice were divided into three groups (3 mice/group) randomly with different treatments: Control group with PBS (Group 1), ICG-ZnS NPs without NIR irradiation (Group 2), and ICG-ZnS NPs with NIR irradiation (Group 3).

### In vivo anti-biofilm activity and wound healing evaluation

The control group was treated with only PBS (200 μL) dripped on the wound. For groups 2 and 3, ICG-ZnS NPs (200 μg mL^−1^, 200 μL) were dropped onto the wound surface respectively, and group 3 was treated with NIR irradiation (1.0 W cm^−2^, 10 min). At the same time, the body weight and wound size were measured after the mice were anesthetized on days 0, 1, 3, 5, and 7 of modeling. After 7 days of treatment, the infected wounds’ tissues in different groups were collected and homogenized in sterile PBS and plate counting assay was applied to quantitatively evaluate the efficiencies of MRSA biofilms elimination. On treatment day 7, some mice were euthanized and skin tissues at the infected wound sites were dissected. MRSA bacterial residues and wound healing in surrounding tissues of various infected wounds were further evaluated. After fixation with 4% paraformaldehyde solution, these skin tissues were stained with hematoxylin and eosin (H&E), Masson’s trichrome (MT), Giemsa, and immunohistochemistry by CD31 staining.

### In vivo anti-inflammation evaluation

Some cytokines involved in inflammatory responses were evaluated on day 7, including tumor necrosis factor-α (TNF-α) and interleukin 6 (IL-6). Briefly, collected skin tissues were fixed with 4% paraformaldehyde solution. After dehydration, the skin tissues were embedded in paraffin and sectioned for TNF-α and IL-6 double immunofluorescent staining. In addition, the inflammatory response in the blood was further evaluated. Blood was collected on day 7 and proinflammatory cytokine expression (TNF-α and IL-6) was evaluated using the corresponding enzyme-linked immunoassay (ELISA) kits.

### In vivo biosafety study

To assessed the biosafety of ICG-ZnS NPs, major organs of mice, including the heart, lungs, liver, spleen, and kidneys, were collected and excised for pathological analysis. All tissue samples were fixed in 4% paraformaldehyde, dehydrated, and embedded in paraffin for sectioning, followed by stained with H&E. In addition, a routine blood test in mice was performed on day 7 to evaluate the potential toxicity of ICG-ZnS NPs.

### Statistical analysis

All assays were repeated at least three times. Data was presented as the mean ± standard deviation (SD) of the minimum number of samples (n ≥ 3). Statistical significance in the mean values was performed using GraphPad Prism 8.0 and SPSS software (version 20.0). *P < 0.05 and **P < 0.01 were considered statistically significant.

## Supplementary Information

**Additional file 1.** pH-responsive hierarchical H_2_S-releasing nano-disinfectant with deep-penetrating and anti-inflammatory properties for synergistically enhanced eradication of bacterial biofilms and wound infection.

## Data Availability

All data generated or analyzed during this study are included in this published article and its supplementary information file.
