# Supplementary material for: pH-responsive hierarchical H2S-releasing nano-disinfectant with deep-penetrating and anti-inflammatory properties for synergistically enhanced eradication of bacterial biofilms and wound infection
Source: J Nanobiotechnology. 2022 Jan 29;20:55. doi: 10.1186/s12951-022-01262-7 (PMC8800305; doi:10.1186/s12951-022-01262-7)
Supplement: Supplementary file 1 — Additional file 1. pH-responsive hierarchical H2S-releasing nano-disinfectant with deep-penetrating and anti-inflammatory properties for synergistically enhanced eradication of bacterial biofilms and wound infection. [file 12951_2022_1262_MOESM1_ESM.docx]

***Additional file 1***

**pH-Responsive Hierarchical H_2_S-Releasing Nano-Disinfectant with Deep-Penetrating and Anti-Inflammatory Properties for Synergistically Enhanced Eradication of Bacterial Biofilms and Wound Infection**

Yue Zhang^1#^, Tianxiang Yue^1#^, Wenting Gu^1^, Aidi Liu^1^, Mengying Cheng^1^, Hongyue Zheng^3^, Dandan Bao^4^, Fanzhu Li^1,2*^, Ji-Gang Piao^1,2,5*^

1. *School of Pharmaceutical Sciences, Zhejiang Chinese Medical University, 310053 Hangzhou, PR China.*
2. *Key Laboratory of Neuropharmacology and Translational Medicine of Zhejiang Province, School of Pharmaceutical Sciences, Zhejiang Chinese Medical University, 310053 Hangzhou, PR China.*
3. *Libraries of Zhejiang Chinese Medical University, Zhejiang Chinese Medical University, 310053 Hangzhou, PR China.*
4. *Department of Dermatology & Cosmetology, The First Affiliated Hospital of Zhejiang Chinese Medical University (Zhejiang Provincial Hospital of Traditional Chinese Medicine), 310053 Hangzhou, PR China.*
5. *Academy of Chinese Medical Science, Zhejiang Chinese Medical University, 310053 Hangzhou, PR China.*

*^#^ These authors contributed equally to this work.*

** Corresponding Author:*

*Ji-Gang Piao, E-mail address: jgpiao@zcmu.edu.cn;*

*Fanzhu Li, E-mail address: lifanzhu@zcmu.edu.cn.*


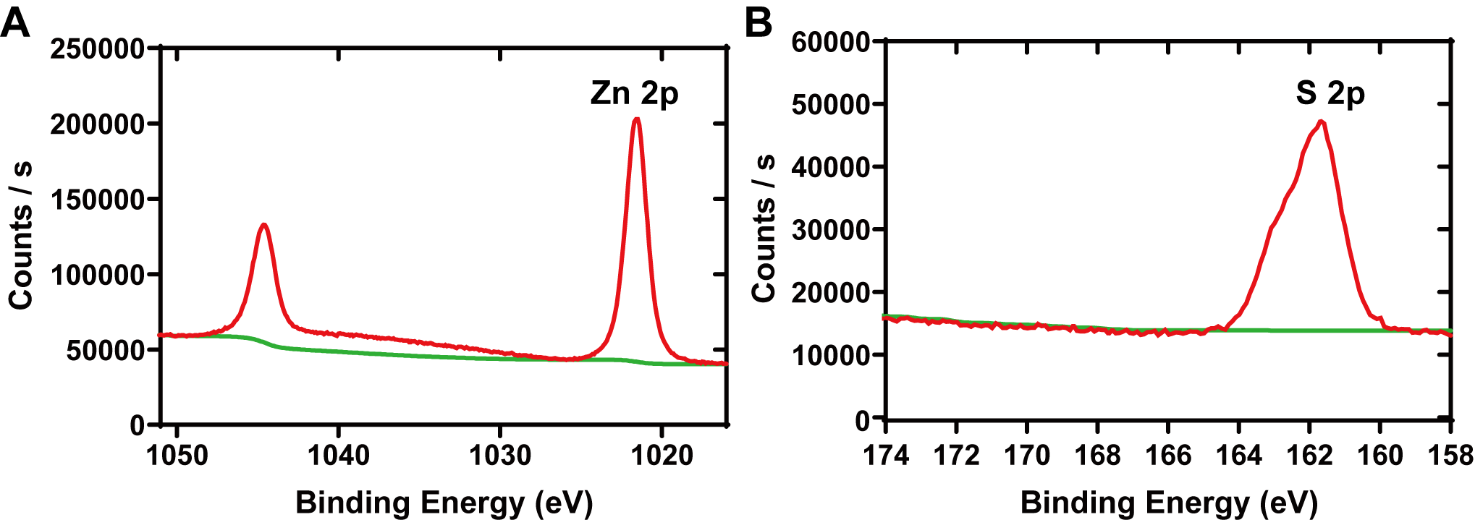


**Fig. S1 (A) Core level XPS spectrum of Zn 2p in ZnS NPs. (B) Core level XPS spectrum of S 2p in ZnS NPs.**

**Fig. S2 Hydrodynamic size distribution of ZnS NPs and ICG-ZnS NPs.**

**A**

**B**

**C**

**Fig. S3 Zeta potentials of ZnS NPs (A), ICG (B)and ICG-ZnS NPs (C).**

**Fig. S4 Standard curve of ICG.**

**Fig. S5 Standard curve of Na_2_S for H_2_S evaluation.**

**Fig. S6 Survival percentage of MRSA treated ICG with irradiated NIR (1 W cm^-2^, 10min).**

**Fig. S7 Survival percentage of MRSA treated ICG in darkness.**
